# Enhancing Healthcare Preparedness: Lessons from a Tabletop Exercise on Highly Pathogenic Avian Influenza (HPAI)

**DOI:** 10.3390/tropicalmed10020047

**Published:** 2025-02-06

**Authors:** Priya Dhagat, Joshua Coan, Ayanava Ganguly, Cole Puetz, David Silvestri, Syra Madad

**Affiliations:** 1 System-Wide Special Pathogens Program, New York City Health and Hospitals, New York, NY 10004, USA; dhagatp@nychhc.org (P.D.); jmcoan@alumni.princeton.edu (J.C.); ag1180@princeton.edu (A.G.); cp3230@caa.columbia.edu (C.P.); 2 Department of Global Health, Princeton University, Princeton, NJ 08544, USA; 3 Department of Epidemiology, Columbia University, New York, NY 11032, USA; 4 Department of Emergency Management, New York City Health and Hospitals, New York, NY 10004, USA; david.silvestri@nychhc.org

**Keywords:** tabletop exercise, Highly Pathogenic Avian Influenza (HPAI), H5N1, healthcare system preparedness, infection prevention and control, early detection and response

## Abstract

Tabletop exercises offer a structured opportunity to assess strengths and potential gaps in preparedness and response plans in a safe learning environment. The New York City Health + Hospitals System-Wide Special Pathogens Program conducted an innovative and multidisciplinary tabletop exercise involving escalating scenarios of highly pathogenic avian influenza (HPAI) H5N1 in 2023. The goals were to assess patient screening processes for infectious diseases within healthcare facilities, infection prevention and control strategies, risk communication, and notification and involvement of public health stakeholders. Participants reflected on previous outbreak responses, discussed the importance of risk communication, and shared insights on tools and resources that would better support healthcare professionals in identifying and managing patients with HPAI/H5N1 infection. Key takeaways included establishing clear protocols for HPAI/H5N1 management, providing timely education to healthcare professionals, and assessing useful communication modalities. Methods: The exercise included escalating scenarios involving a single case of HPAI/H5N1 advancing to community transmission. Key participants spanned clinical departments, infection prevention and control, emergency management, and local public health stakeholders. Structured discussions targeted triggers for escalation, infection prevention strategies, and communication pathways. Results: Findings highlighted the need for tailored screening criteria, robust infection prevention protocols, clear communication strategies, and collaboration with public health authorities. Specific improvements included refining triage and isolation protocols, enhancing staff education, and leveraging syndromic surveillance systems. Conclusion: This exercise underscored the importance of proactive planning, multidisciplinary collaboration, and integration of biosafety measures to strengthen healthcare system resilience against HPAI/H5N1.

## 1. Introduction

Influenza viruses are constantly evolving due to genetic mutations to surface proteins HA (hemagglutinin) and NA (neuraminidase) that occur during viral replication, posing a significant biosecurity threat and impacting public health. Influenza viruses circulate year-round, but certain strains of Alphainfluenzavirus influenzae and Betainfluenzavirus influenzae (i.e., Influenza A and B) surge throughout the winter season in the United States, spreading easily between humans leading to illness and potentially severe complications. Annual flu vaccination can prevent disease caused by circulating Influenza A and B strains. Highly pathogenic avian influenza (HPAI) is a respiratory illness caused by avian-origin influenza A virus subtypes, such as H5, H7, and H9 [1,2]. Influenza A viruses infect the respiratory and gastrointestinal tract of birds, leading to virus shedding in saliva, mucus, and feces, potentially causing infection in humans through exposure to infected live or dead poultry or contaminated environments [1,3]. Human infections with A(H5N1), A(H7N9), and A(H9N2) were initially reported in China in 1997, 2013, and 1998, respectively [4]. More recently, instances of H5N1 influenza have been reported in numerous marine mammals in South America, and significant outbreaks have occurred among wild birds, domestic poultry, and dairy cattle in the United States, leading to the culling of over 58 million birds and affecting over 900 dairy herds nationwide [5,6,7]. The first recorded instance of HPAI in a human within the United States was documented on 28 April 2022 [8]. Since 2024, a majority of human cases in the United Stated have been associated with exposure to or direct contact with sick or dead poultry and infected dairy cattle, underscoring the increasing spillover of H5N1 to humans. During the time of the HPAI/H5N1 tabletop exercise in 2023, only wild birds, poultry flocks and other mammals were infected. However, at the time of publication of this article the ongoing, multi-state outbreak among U.S. dairy cows has been reported. The outbreak among dairy cattle in the U.S. was documented to have started in March 2024 [9]. This is a relatively new and alarming phenomenon, as H5N1 was not previously documented in cattle to this extent, making it a cause for concern among the public health community. The virus can be present in respiratory secretions and raw milk from infected cows, posing a potential risk if consumed unpasteurized.

H5N1 influenza virus infection in humans can cause a range of symptoms, from mild upper respiratory tract symptoms (e.g., fever, cough, sore throat, runny or stuffy nose, muscle aches, headaches, fatigue, and shortness of breath) to lower respiratory tract disease. Infection can progress to serious complications such as severe pneumonia with respiratory failure, encephalitis, and multi-organ failure [10]. Recent human cases of H5N1 in the United States from March 2024 to October 2024 revealed the following symptoms among adults exposed to infected poultry or dairy cows: 93% had conjunctivitis (eye redness), 49% fever, and 36% respiratory symptoms. The historic mortality rate of H5N1 infection in humans is approximately 50% [11]. There have been over 860 human infections with H5N1 bird flu viruses in over 20 countries reported to the WHO between 2003–2022, but the risk for sustained human transmission is thought to be low, considering H5N1 viruses do not have the ability to easily bind to receptors in the upper respiratory tract of humans to become highly transmissible [12]. Despite the low risk, the potential for influenza viruses to evolve rapidly, combined with their widespread presence in wild birds and poultry, suggests that isolated human infections may continue.

In fact, the alarming increase in mammal infections globally lead to a joint statement from the UN Food and Agriculture Organization (FAO), the World Health Organization (WHO), and the World Organization for Animal Health (WOAH) in 2023, which urged countries to increase surveillance for avian flu viruses in poultry farms, citing that H5N1 clade 2.3.4.4b was reported in 67 countries across 5 continents in 2022 and 14 more countries in 2023, mainly in the Americas [13]. Therefore, rigorous monitoring of these viruses in birds, poultry, mammals, and humans across the globe, along with periodic risk evaluations and sustained readiness, is imperative.

In light of the persistent poultry and dairy cattle outbreaks, alarming rise in mammal infections, and growing number of human cases domestically and globally, healthcare systems must remain vigilant and ready to swiftly identify and address cases involving symptoms or exposure to HPAI/H5N1. The dense and diverse population of New York City within New York state, a leading agriculture state, planning for the care and management of human cases and strengthening preparedness is critical. The New York City Health and Hospitals Corporation (NYC Health + Hospitals) is the largest municipal health care system in the United States, providing care to New Yorkers across the city’s five boroughs. In an effort to prepare for potential cases of HPAI/H5N1 in a healthcare setting, a tabletop exercise was conducted by the New York City Health + Hospitals System-wide Special Pathogens Program in which participants representing various clinical departments from frontline acute care hospitals, including representatives from adult and pediatric emergency departments, intensive care units, infection prevention and control (IPaC), laboratory services, pharmacy, supply chain, occupational health services, and emergency management as well as representatives from the local public health department discussed planning and response measures when facing varying levels of a hypothetical HPAI/H5N1 outbreak, starting with a single case and escalating to a widespread community outbreak via human-to-human transmission. To understand current processes, the exercise focused on three objectives: (1) identifying key triggers and efforts to support proactive systemwide escalation of an HPAI/H5N1 outbreak response; (2) reviewing processes to minimize transmission within healthcare facilities and prevent exposure to staff and patients; and (3) discussing strategies to establish, maintain, and track communication with staff and situational awareness.

## 2. Methods

### 2.1. Format

To set the stage and provide a comprehensive overview, participants were first introduced to an informational session. This session covered fundamental epidemiological concepts, including modes of transmission, incubation periods, clinical presentations, virulence factors, and case fatality rates. Following this, participants engaged in a series of three progressively challenging modules, each simulating an escalating outbreak scenario. After each module, a structured discussion took place, targeting specific departments within healthcare facilities and the local public health department (Table 1). These discussions aimed to critically evaluate preparedness, response capabilities, and communication strategies from an emergency management perspective. The tabletop exercise was led by an infectious disease epidemiologist and an infection preventionist. Participants joined in a hybrid format, in a conference room as well as joining virtually. A total of less than 40 participants across the health system and local health department joined for this half day tabletop exercise.

### 2.2. Modules

Module 1 initiated the discussion with a case involving a 50-year-old male from New York who was exposed to an infected poultry flock. The individual sought medical care after experiencing symptoms including fever, cough, myalgia, and shortness of breath.

Module 2 intensified the scenario by incorporating the patient’s family members, one of whom was a child. These new patients, displaying symptoms akin to the first case, sought treatment at the hospital. The situation worsened as the index patient’s condition deteriorated, necessitating intensive care.

Module 3 further heightened the stakes by involving healthcare workers, who became exposed to the patients from the previous modules. This module also depicted an increase in the number of patients presenting with influenza-like illness (ILI), indicating a broadening of the outbreak’s impact.

Facilitator Injects and Discussion: Each module began with a scenario introduction, followed by structured questions to elicit discussion on triage protocols, infection control measures, diagnostic testing, and communication strategies. Facilitator injects guided participants in addressing evolving challenges. The discussions also evaluated the availability and adequacy of personal protective equipment (PPE), negative pressure rooms, and protocols for disinfection and waste management.

Data Collection: Key insights were documented during structured discussions and the subsequent hotwash session. These were analyzed to identify actionable recommendations for enhancing preparedness and response, with an emphasis on integrating lessons learned from previous outbreaks like COVID-19 and mpox.

## 3. Results

Module 1: The initial manifestation of HPAI/H5N1 can closely resemble other infections, including seasonal influenza and COVID-19. Consequently, Module 1 emphasizes the identification of pivotal indicators that necessitate an escalated response from healthcare facilities. In this context, consensus was reached among all participants, especially those from the emergency department, on the necessity and implementation of robust early detection tools. As the potential for HPAI transmission to humans increases, incorporating a specific query about poultry/cattle exposure during initial patient evaluations (e.g., during triage) is considered optimal. Participants further highlighted the importance of a clear definition of “exposure”, advocating for staff to be well-informed about these definitions to avoid broad interpretations. Integrating HPAI/H5N1 exposure criteria, as outlined by CDC guidelines, into screening questionnaires can help healthcare staff capture extensive exposure-related information at the outset of the visit. Meeting any of these exposure criteria necessitates awareness of the existing, clearly defined chain of command, akin to that established for other significant infectious diseases, which includes notifying department leadership and the local health department.

To facilitate implementation, the following strategies were prioritized:

- Integration of exposure criteria into electronic health records (EHR) and screening questionnaires.
- Staff training to ensure accurate understanding of case and exposure definitions and workflows.
- Establishment of detailed triage workflows to guide the early detection and isolation of suspected HPAI/H5N1 cases.

Module 2: As the situation intensifies in Module 2, with both pediatric and adult patients presenting with ILI and a relevant epidemiological link to the initial patient, participants demonstrated a profound understanding of crucial infection prevention and control measures required to manage the patients effectively. These measures include implementing appropriate transmission-based precautions, isolating the patient in a negative pressure room, and utilizing dedicated equipment for their care. An innovative suggestion from clinical staff was the formation of a dedicated team to manage suspected HPAI/H5N1 patients, minimizing staff exposure. Given that most hospital clinical laboratories conduct rapid influenza A or B tests but not necessarily subtyping, immediate collaboration with local public health authorities for guidance on specimen collection and subtyping at public health laboratories was unanimously endorsed. Discussions on immediate antiviral treatment reflected a high level of confidence, with clinical participants acknowledging CDC recommendations to commence treatment promptly, complemented by supportive care. Building on the experience from COVID-19 and mpox, healthcare facilities have established efficient contact tracing protocols, which would be adhered to in this scenario. In situations posing a high risk of community transmission, leveraging telehealth was emphasized as a strategy to prevent emergency department overloads.

To facilitate implementation, the following strategies were prioritized:

- Placing patients in negative pressure rooms and implementing standard, contact, and airborne precautions with eye protection.
- Formation of dedicated care teams to minimize staff exposure while ensuring patient management efficiency.
- Utilizing rapid Influenza A/B testing, with immediate collaboration with local health authorities for specimen subtyping at public health laboratories or reference laboratories.
- Environmental cleaning and disinfection measures to reduce contamination risks, especially in waiting rooms, patient-care areas, and frequently touched surfaces [14].
- Maintaining a clear chain of command for decision-making during outbreaks.
- Leveraging telehealth services to prevent emergency department overload of patients.
- Proactively managing personal protective equipment inventory and/or stockpiling supply.

Module 3: Effective and continuous communication between staff and leadership is pivotal for maintaining situational awareness and for the efficient coordination and escalation of operations when required. In Module 3, the scenario delved into potential local community transmission, emphasizing strategies to initiate and sustain communication with staff. Participants referenced existing communication channels at both the facility level (e.g., department-specific notification protocols) and the system level (including all-staff emails, emergency alerts, and dedicated employee intranet websites). Beyond identifying these communication modalities and tools for situational awareness, the discussion unveiled the necessity for a predefined threshold of cases, which, once reached, would trigger notification of all system staff regarding suspected HPAI/H5N1 cases. This decision is heavily reliant on guidance from public health partners. Nonetheless, there was unanimous agreement on the importance of timely staff education about HPAI/H5N1, particularly regarding exposure and screening criteria. Furthermore, the deployment of customized signage and posters at entry points can assist in prompting patients to self-report exposure. Key insights from the exercise are summarized in Table 2.

To facilitate implementation, the following strategies were prioritized:

- Real-time updates via EHR-integrated syndromic surveillance dashboards and system-wide emergency alerts.
- Establishing predefined thresholds for notifying staff about suspected HPAI/H5N1 cases, developed in collaboration with public health partners.
- Utilizing diverse communication channels, such as department-specific notifications, staff-wide emails, intranet portals, and text alerts.

## 4. Discussion

The integration of the “identify, isolate, inform” paradigm in healthcare settings, especially through tabletop exercises, has consistently proven pivotal in enhancing the readiness and response to infectious diseases like HPAI [15]. These exercises, serving as a critical tool in emergency preparedness, offer a strategic platform to assess, review, refine and validate protocols and processes in a pragmatic, risk-free environment, ensuring that healthcare systems are well-equipped to manage infectious threats effectively [16]. They not only fortify the healthcare system’s preparedness and response mechanisms but also instill a culture of continuous learning and adaptability.

By incorporating the “identify, isolate, inform” approach into a tabletop exercise, participants were challenged to think back to early encounters of previous outbreaks, such as COVID-19 and mpox, and lean on lessons learned to tailor processes where needed. Specific screening criteria, diagnostic testing, treatment, and hospital admission criteria may vary greatly, and, for HPAI, it has become apparent from this exercise that healthcare staff must have resources readily and easily accessible for prompt refreshers on exposure criteria. Without the knowledge on how to screen for HPAI, testing and treatment can be delayed and exposures to staff and others may occur. Ongoing communication is essential —for early notification but also for situational awareness—to establish and maintain awareness among the healthcare workforce.

Each of the 3 modules of the tabletop exercise represented a different scenario that tested a healthcare facility, and its larger public health system, in terms of their readiness. While it was the NYC Health + Hospitals under review in this tabletop, the key takeaways could inform and improve the actual responses of any healthcare system, which is where the value of such an exercise lies. Future related exercises should include the updated epidemiology of H5N1 and transmission of this virus among multiple animal species, including dairy cattle within the methodology. Furthermore, using the updated public health case definition of H5N1 for screening purposes is essential. For the United States, the H5N1 epidemiological criteria includes persons with exposure to infected birds or other animals through close contact or direct contact with contaminated environments, products or equipment, exposure to a suspected of confirmed person with H5N1, or laboratory exposure [17].

In line with this focus on the “identify” step of the special pathogens response, the key limitation in this tabletop exercise is the lack of discussion surrounding the pan-zoonotic dimension of HPAI. The participants focused heavily on suspected human cases but considering that the current US HPAI outbreaks are all primarily in poultry and dairy cattle, attention should also be given to monitoring these animal populations, and as a corollary, the human populations that interact with them. The Unites States Department of Agriculture, as well as the New York State Department of Agriculture and Markets, maintains active lists and a dashboard of HPAI detections in commercial and backyard flocks; thus, with the support of local public health departments, veterinarians and veterinary epidemiologists, the tracking of these types of outbreaks in animal populations is vital to identifying when HPAI may mutate and allow the virus to infect humans [18,19,20]. Using this data and support from public health can then allow healthcare systems to clearly define a threshold at which their facilities are notified about the possibility of human HPAI cases entering their facility. This will help providers prepare to see the first case of HPAI in their emergency department before animal-to-human, or human-to-human transmission.

Moreover, integrating veterinary data into innovative tools like syndromic surveillance dashboards for healthcare systems can further enhance the early detection capabilities and provide early warning signs in addition to bolstering early identification processes and allowing for a rapid and informed response to emerging infectious threats like HPAI. This syndromic surveillance platform could be incorporated into a health system’s electronic health records system and be available to anyone involved in the patient identification process, from registration staff to healthcare providers. This would support and allow for monitoring of ILI or other symptoms, and trigger an escalation in response. In addition, this type of syndromic surveillance system could be an essential tool for healthcare systems to monitor the symptom prevalence in local communities in general, which would also help supplement and direct preparedness efforts.

## 5. Conclusions

This paper highlights the invaluable role of tabletop exercises, offering a structured opportunity to identify potential gaps in emergency protocols and communication strategies, ensuring a swift and coordinated response to actual outbreaks, and ultimately helping fortify healthcare systems against infectious diseases like HPAI. By simulating various outbreak scenarios, healthcare professionals can scrutinize and enhance their response strategies, ensuring rapid adaptability and effective management of real-world outbreaks. As the threat landscape of HPAIs continues to evolve, such exercises are indispensable in fostering a proactive, informed, and resilient healthcare infrastructure. However, for a holistic approach to infectious disease readiness, it’s crucial to extend the focus beyond human cases to include the monitoring of animal populations and the integration of innovative digital tools for early detection. These comprehensive measures ensure that healthcare systems are not just reactive but proactive in their approach to managing infectious diseases, ultimately safeguarding public health and mitigating the risk of widespread outbreaks.

## Figures and Tables

**Table 1:** Examples of Facilitator Questions.

Module	Department	Facilitator Questions
Module 1	Emergency Department	At this point in the scenario, what would your next step be in clinical care of this patient? Are there additional tests you would consider?
		Are there any infection prevention measures you would implement at this point? Would you place this patient in isolation? What types of transmission-based precaution/PPE would you use, if any?
		Would you want updates on the HPAI incidence in NYS? If so, how would you like to receive this information?
	Communications/Marketing	With the increased media coverage about HPAI in NYS, is there a need to communicate the threat level of HPAI to patients and staff? What kind of information would be most helpful for staff to be aware of for human cases of HPAI? Or, would you wait until the NYC DOHMH shares information?
Module 2	Infection Prevention, Infectious Disease	After becoming aware of the patient’s exposure to birds, what would your next steps be? What actions should be considered to prevent transmission within the ICU (room type, PPE, transmission-based precautions, etc)?
	Laboratory	What is the process to coordinate specimen collection/send out for subtyping?
	Local Public Health Department	What would be the turnaround time for testing/subtyping? What guidance would be given to healthcare providers?
	Emergency Management/ Communications/Marketing	When should information about a suspected case of HPAI be shared with staff? Who should be notified (system leadership, facility leadership, facility staff, all staff)? What types of communication methods will be used (e.g., email to staff; FAQs for staff)?
	Supply Chain/Pharmacy	Are there any considerations with procuring an increased amount of anti-viral medication? What factors may influence this decision?
	Occupational Health Services	What is the process for contact tracing? Would there be any changes to existing protocols?
	All Participants	What types of guidance’s should be developed, in collaboration with other departments? What other considerations are you or your units having at this point, or should we be considering at the system level?
Module 3	Emergency Department	What kind of decision support tools would be helpful for screening patients if HPAI incidence increases?
	Emergency Management	As we anticipate the need for increased testing, what actions, teams, resources, and space are needed to support?

**Table 2:** Key Takeaways from the HPAI/H5N1 Tabletop Exercise.

Module/Scenario	Objective	Key Takeaways
Module 1: A 50-year-old male from New York had exposure to an infected flock in New York and presented to a hospital with fever, cough, myalgia, and shortness of breath.	Identify pivotal triggers and strategize for a proactive, system-wide escalation in response to an HPAI outbreak.	Determine when HPAI specific screening criteria should be implemented during registration or triage by referring to incidence rates and available data in conjunction with guidance from public health partners. Provide timely education to staff about exposure definitions and epidemiologic linkages specific to HPAI. Assess which communication methods and modalities are most useful to reach staff if system-wide notifications for situation awareness are required.
Module 2: The family members of the index patient, including a child, presented to the hospital with similar symptoms. The initial patient requires intensive care support due to the severity of illness.	Review processes to minimize transmission within healthcare facilities and prevent exposure to staff and patients	Ensure facility staff are aware of notification protocols to escalate as needed. Ensure healthcare workers are educated and have refresher trainings on infection prevention and control measures. Establish clear processes for influenza A subtyping, whether conducted in-house or at a reference laboratory. Ensure that disease mitigation strategies and communication channels are standardized across healthcare facilities in the entire system. Utilize telehealth appointments as much as possible using appropriate messaging to patients on exposure criteria and when to seek medical care to prevent overcrowding at emergency departments.
Module 3: Following a confirmed suspicion of HPAI, healthcare workers have been exposed to patients from Modules 1 and 2, with an increasing number of patients exhibiting ILI symptoms.	Discuss strategies to establish, maintain, and track communication with staff and situational awareness	Collaborate with public health partners on outbreak response strategies, send-out testing, anti-viral medication stockpiling, and public messaging. Ensure plans, protocols, and guidance are in place and relevant parties have clear understanding. Establish a workgroup and planning team with key stakeholders across the healthcare system as well as public health partners. Leverage lessons learned, former processes, and existing surge space as needed.

## Data Availability

The original contributions presented in this study are included in the article. Further inquiries can be directed to the corresponding author.
